# Environmental Working Conditions, Lung Function and Total Serum Bile Acids of Spray Painters Exposed to Organic Solvents in Ile-Ife, Nigeria

**DOI:** 10.5696/2156-9614-7-13.2

**Published:** 2017-03-29

**Authors:** Temitope Olumuyiwa Ojo, Adedeji A Onayade, Patrick Ayodeji Akinyemi, Adewole J Adesanmi

**Affiliations:** 1 Obafemi Awolowo University Teaching Hospital Complex, Ile-Ife, Osun State, Nigeria; 2 Department of Community Health, Obafemi Awolowo University, Ile-Ife, Osun State, Nigeria; 3 Department of Chemical Engineering, Obafemi Awolowo University, Ile-Ife, Osun State, Nigeria

**Keywords:** spray painters, volatile organic compounds, lung function, total serum bile acids, workshops, Nigeria

## Abstract

**Background.:**

Nigeria has a growing spray painting industry, however, the burden of occupational health problems related to organic solvent exposure among spray painters in Nigeria is under-studied.

**Objectives.:**

This study aimed to assess workshop characteristics and ambient concentration of total volatile organic compounds (VOCs) in spray painting workshops and to compare lung function status and total serum bile acid levels of spray painters and controls.

**Methods.:**

A cross-sectional study design was employed to survey 120 spray painters and 120 controls (electronic technicians). A semi-structured questionnaire was used to obtain data on socio-demographics characteristics of the respondents. Weight, height and lung function of respondents were measured. In addition, a checklist was used to survey the spray painting workshops. Total VOC levels were determined in 37 spray painting and 31 electronic workshops. Data were analyzed using Statistical Program for the Social Sciences (SPSS) version 20 and a p-value of <0.05 was considered to be statistically significant.

**Results.:**

Windows were present in only 5 (13.5%) spray painting workshops and 23 (62%) workshops had a retractable tarpaulin at the entrance. Only 9 (24%) workshops had changing rooms, while fire extinguishers and first aid kits were not present in any of the surveyed workshops. A respirator with filter was sighted in only 1 (3%) workshop. The 8-hour time weighted average concentration of total VOCs in spray painting workshops was 13.4 ppm, which is above the national permissible exposure limits of 1.9 ppm. Forced vital capacity (FVC) percent predicted was significantly lower in spray painters (93.9 ±10.8%) than controls (96.7± 8.2%) (t = −2.326, df=238 p< 0.001). In addition, forced expiratory volume in the first second (FEV1) percent predicted was lower in spray painters (94.6±12.2%) than controls (100.3±9.1%) (t=−4.058, df=238, p=0.002). FEV1/FVC% was significantly lower among spray painters (85.48±8.70%) compared with controls (87.88±6.22%) (t=−2.861 df=238, p= 0.005). Total serum bile acids was significantly elevated in painters (8.71±3.39 mmol/l) compared to controls (4.67 ±2.15 mmol/l) (t=10.358, df=213, p<0.05).

**Conclusions.:**

Spray painters in the present study conduct their activities in hazardous work settings. More needs to be done concerning workplace regulation and enforcements to ensure that spray painters comply with minimum standards of occupational safety, workplace hygiene and sanitation.

**Patient Consent.:**

Obtained

**Ethics Approval.:**

Ethical approval was granted by the Health Research and Ethics Committee of the Institute of Public Health, Obafemi Awolowo University.

## Introduction

Nigeria has a growing spray painting industry, as it has one of the highest number of automobiles in Africa, estimated at around 14.6 million as of 2015.^1^ Most auto painting in Nigeria is done in workshops and sheds that do not have specialized spraying booths.^2^ Spray painters work mainly in the small- and medium-scale sectors of the Nigerian economy and it is a vocation characterized by exposure to chemical hazards, as auto workshops are estimated to generate the highest proportion of small-volume hazardous wastes in the country.^3^ The painting procedure itself offers the highest degree of exposure as the paint is aerosolized and spread on part of the automobile. Likewise, the period of allowing the paint to dry on the painted parts of the automobile also releases some of the organic solvents in the form of volatile organic compounds (VOCs).

Since these vapors can be inhaled or absorbed through the skin, individuals who perform this task and do not wear appropriate, personal protective equipment (PPE) such as a respirator or gloves can be adversely affected.^4^,^5^ Furthermore, considerable inhalation and percutaneous absorption of solvents can occur within minutes of the onset of exposure. Most painting workshops are poorly ventilated and painters often totally enclose their spraying areas during and just after the painting process with make-shift items such as tarpaulin to prevent dust from settling on painted surfaces, and this method exposes spray painters to extremely high levels of chemical hazards.^6^

The adverse health effects of organic solvents can be classified according to respiratory, neurotoxic and dermatologic effects. Other less common ill effects include eye irritation and liver and kidney damage. Decreased lung function parameters and the high prevalence of asthma symptoms have also been documented in past studies.^7^,^8^ A study conducted among workers in a paint production factory in Lagos, Nigeria demonstrated that PPE use was generally low and that workers had higher urine concentrations of heavy metals than controls.^8^ This same study observed that 90% of workers reported at least one work-related health symptom. Another study that assessed liver and renal function among paint factory workers in Anambra State revealed that paint factory workers had elevated liver enzymes and higher serum electrolytes concentrations than controls.^9^ A lung function assessment among foam industry workers in Onitsha, Anambra State, Nigeria reported lower peak flow readings among workers with presumed exposure to organic solvents compared to controls.^10^ In a study conducted in Calabar, Nigeria, spray painters had reduced lung function compared with controls, although solvent exposure was not assessed and the selected controls were from different socioeconomic backgrounds from the spray painters.^2^

**Abbreviations**
*FEV1*: Forced expiratory volume in the first second
*FVC*: Forced vital capacity
*PPE*: Personal protective equipment
*TSBA*: Total serum bile acids
*VOC*: Volatile organic compounds

Overall, the burden of occupational health problems related to organic solvent exposure among spray painters in Nigeria remains largely unquantified. Permissible exposure limits for solvents across workshops are neither readily available nor enforced in Nigeria. Moreover, spray painters work with these hazardous chemical agents which are often mishandled due to poor availability and use of PPE.^11^ This poses serious health risks to the workers themselves and the immediate environment around their workplaces. Solvent exposure has been associated with various health conditions including occupational asthma, and few studies have been done in Africa in this area of occupational epidemiology.^12–14^ Biomarkers can be used to assess exposure to organic solvents and its effects on various organ systems of the body. Total serum bile acids (TSBA) is a useful biomarker and has been reported to be better than the liver enzyme test as a biomarker of solvent-induced hepatotoxicity.^15–17^ It is also cost-effective, which makes its use pragmatic in resource-constrained settings like Nigeria. Pulmonary function assessments of welders, flour mill workers and sawmill workers have been performed in Nigeria, but spray painters have been sparsely studied.^18–24^ This study assessed lung function and total serum bile acid status among spray painters and controls in Ile-Ife Nigeria. The findings from this survey will guide policy making and appropriate interventions to improve the knowledge of these artisans about the hazards of their occupation and optimize healthy work practices to ensure their health and wellbeing.

## Methods

### Study Location

This study was conducted in Ile-Ife, one of the biggest towns in Osun State, Nigeria. The town has two local government areas with a population size of over 328,000, as reported in the 2006 national population census.^25^ Most inhabitants are farmers, artisans, civil servants, and traders. Spray painters usually occupy partly developed and undeveloped plots of land, while electronic technicians work individually or in small groups in shops or makeshift sheds. Spray painters and electronic technicians' workshops were widely distributed in the metropolis.

### Study Design and Population

A cross-sectional study design was employed. One-hundred and twenty painters (60 masters and 60 apprentices) were selected using a table of random numbers from a sampling frame of 96 registered members and 76 eligible apprentices. Similarly, 120 electronic technicians (60 masters and 60 apprentices) were recruited. Sixty master technicians were selected from a sampling frame of 158 members, while 60 apprentices were selected from a list of 92 eligible apprentices. Controls were selected in such a way so as to match the characteristics of the selected spray painters. All the workers were males. The control and study groups were matched for age, weight and height on a group basis using frequency matching techniques. For participants to be included in the study, they must have engaged in the vocation for at least one year. Respondents who were hospitalized in the last one month or those with neuromuscular disease, underlying lung disease such as tuberculosis and those with abnormalities of the vertebra column as well as thoracic cage, e.g. kypho-scoliosis, were excluded.

Ethical approval was granted by the Health Research and Ethics Committee of the Institute of Public Health, Obafemi Awolowo University. Written consent was obtained from all adult participants. For participants less than 18 years of age (apprentices), assent was obtained from the participants, consent was obtained from their parents or guardians and permission was obtained from their supervisors.

### Sample Size Determination

The minimum sample size was determined using the following formula for calculating sample size for the comparison of means of two independent groups (*Equation 1*):^26^

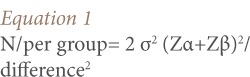



Where N/per group = minimum sample size per group and Zα = standard normal deviate of α at 95% confidence level = 1.96, Zβ = standard normal deviate of β at 80% confidence level = 0.84 while difference = mean difference in forced expiratory volume between the two groups that the investigator is willing to accept (0.19 liters), and σ = standard deviation of the forced expiratory volume in the first second (FEV1) (i.e Sp, the pooled estimate of the standard deviation in the two groups = 0.48 L as extrapolated from findings in the study by Aribo and Antai).^2^

N/per group = 2×0.48^2^ (1.96+0.80)^2^/(0.19)^2^, N/per group = 100. The correction for anticipated attrition of 20% = 20, and therefore the adjusted sample size = 120 each in both spray painters and controls.

### Data Collection

Data were collected from October to December 2016. None of the electronic workshops were located close to spray painting workshops. Data were collected with the aid of an interviewer-administered questionnaire. Information obtained included socio-demographic characteristics as well as medical and occupational history of respondents. A section of the questionnaire was used to record anthropometry such as weight, height and spirometry readings. The questionnaire was translated into Yoruba, which is the local language in the study area and back-translated to English. All subjects were examined privately on site. Measurement of standing height was performed with the aid of a stadiometer which is comprised of a horizontal platform, vertical perpendicular rule and a sliding head board. Subjects were barefooted and stood chest out and erect against the vertical rule with their feet together. Their heels, buttocks and occiput were touching the rule simultaneously while looking horizontally straight ahead. The investigator's eyes were placed level with the headboard to avoid parallax error. Measurements were to the nearest centimeter after sliding down the headboard. All study participants were weighed wearing light clothing and barefooted. Weights were measured using a Camry® weighing scale. The scale was standardized daily before use. Blood pressure was measured using an Accouson's mercury sphygmomanometer (Kris-Alloy, England). Blood pressure was measured in a sitting position with an appropriate cuff size on the left arm.

### Spirometry

Pulmonary function tests were conducted using the Spirolab III (MIR Italy). All spirometry tests were conducted within a fixed period (9am–2pm local time) to minimize diurnal variation.^20^ Spirometry was performed with participants standing relaxed in front of the apparatus without using a nose clip and with all tight clothing loosened. Before starting the test, the procedure was explained to participants. Subsequently, subjects performed forced expiratory maneuvers from total lung capacity to residual volume to obtain measurements of forced vital capacity and forced expiratory volume in the first second. The best readings of forced vital capacity (FVC) and FEV1 were taken from three technically satisfactory forced expiratory maneuvers. Corrections for body temperature and pressure saturated with water vapor were automatically made for all measurements by the device software. Acceptability criteria were considered according to the American Thoracic Society/European Respiratory Society taskforce recommendations.^27^ The European Respiratory Society spirometry reference values adjusted for subjects of African ethnicity as programmed on the Spirolab III device were used for the survey.^28^ Obstructive impairment was established when FEV1/FVC X 100 was < 70%, while restrictive impairment was assumed if FVC was less than 80% of predicted.

### Ambient Concentrations of Total Volatile Organic Compounds

The E8500 plus (E instruments Philadelphia, USA) was used to assess total VOC concentration. The device works through a photoionization detection mechanism and it is sensitive and capable of detecting total VOCs in the range of 0–200 ppm with a resolution of 1 ppm. Before beginning the air sampling each day, electronic calibration of the instrument was carried out according to the manufacturer's instructions. Individual VOCs could not be quantified as the device only estimated total VOC concentration.

Ambient air concentration was assessed when major spraying of a vehicle was carried out. For this study, major spraying occurred when total car body spraying was performed or when at least half of the total surface of a vehicle was painted. Six consecutive readings were recorded over a 30 minute period with the gas sampler placed on a raised platform to correspond to the breathing zones of the workers as much as possible. The average of the 6 readings in parts per million was used to determine an 8-hour time weighted average. Ambient total VOC concentration above 1.9 ppm was considered to be hazardous, since this is the permissible limit in line with the national recommended ambient air quality standards.^29^

### Assessment of Total Serum Bile Acids

Total serum bile acids was assessed by collecting fasting blood samples (early morning without eating) and before commencing the day's work among both spray painters and controls. About 4 ml of venous blood from the cubital vein was withdrawn into plain bottles. Thereafter, the samples were stored in a freezer at −20^°^ C for analysis in batches. Laboratory analysis was done using high performance liquid chromatography.

### Data Analysis

Data were analyzed using SPSS version 20.0. Mean and standard deviation were used to summarize continuous variables such as age, height, income, FVC, FEV1, and FEV1/FVC, while categorical variables were summarized using frequencies and proportions. Student's t test was used to assess difference in means across the study and control groups. The chi-square test was used to assess the relationship between categorical variables, e.g. smoking status and abnormal spirometry results. Likelihood ratio was used to assess the relationship between categorical variables when more than 25% of the cells had an expected count less than 5. A p-value of <0.05 was considered to be statistically significant.

## Results

Socio-demographics characteristics of spray painters and controls are shown in Table 1.

**Table 1:** Socio-demographic Characteristics of Spray Painters and Controls

Study factor	Spray painters N=120	Controls N=120	*p-value*
Age (mean±SD), yrs	32.68 ±13.84	33.87±15.48	0.530
Height in meters (mean±SD), m	1.68±0.06	1.69±0.05	0.478
Weight (mean±SD), kg	62.36±9.97	64.23±10.79	0.163
Years of work experience (mean±SD)	12.77±13.72	13.67±14.27	0.609
Average monthly income (mean±SD)	19867±23970	18375±21003	0.621
Current smokers n (%)	2 (1.7)	4 (3.3)	
Ex-smokers n (%)	12 (10.0)	22 (18.3)	0.112
Non-smokers n (%)	106 (88.3)	94 (78.3)	

Abbreviations: SD, standard deviation

The spray painters and controls were similar in comparable age, height and weight. In addition, years of work experience, monthly income and smoking status did not differ significantly between the two groups.

The physical characteristics of 37 spray painting workshops were assessed and the results are shown in Table 2.

**Table 2:** Physical Characteristics of Spray Painting Workshops, Ile-Ife 2016

**Characteristics**	**Frequency n=37**	**Percentage**
**Workshop size** (m^2^)		
<22.3	10	27.0
≥22.3	27	73.0
**Workshop height**		
≤2.7 m	4	10.8
2.71–4 m	33	89.2
**Workshop wall material**		
Wood	17	45.9
Corrugated iron sheet	9	24.3
Tarpaulin	5	13.5
Brick	3	8.1
None (open shed)	3	8.1
**Workshop floor**		
Bare earth	36	97.3
Concrete	1	2.7
**Ceiling type**		
None (plain roof)	36	97.3
Concrete	1	2.7
**Availability of windows**		
Yes	5	13.5
No	29	78.4
Open shed	3	8.1
**Retractable tarpaulin on the entrance**		
Yes	23	62.2
No	14	37.8

Twenty-seven (73%) workshops had a total area ≥22.3 m^2^, while about 33 (89.2%) of the workshops had a height greater than 2.7 meters. Thirty-six (97.3%) of the workshops had bare earth for a floor and 97.3% of workshops had no ceiling (plain roof). Twenty-nine out of the 37 workshops (78%) had no windows, while 62.2% of workshop entrances used retractable tarpaulin. The walls of 17 (45.9%) workshops were made of wood, while 9 (24.3%) were made of corrugated iron sheeting.

Workshop sanitation and hygiene facilities across spray painting workshops are shown in Table 3. A changing room was available in 9 (24.3%) workshops and only 1 (2.7%) workshop had an eating area. Thirty workshops (81.1%) had no toilets and flammable solvents were properly stored in only 5 (13.5%) workshops. Open dumping and burning was the method of refuse disposal in 33 (89.2%) shops. Fire extinguishers, first aid kits and waste bins were not available in any of the workshops. Nose masks were available in 7 (18.9%) workshops, while safety goggles, face shield gloves, respirators and hearing protectors were not available in any of the workshops.

**Table 3:** Housekeeping, Waste Disposal, and PPE Availability in Spray Painting Workshops

**Characteristics**	**Frequency n=37**	**Percentage**
**Changing room**		
Yes	9	24.3
No	28	75.7
**Separate eating area**		
Yes	1	2.7
No	36	97.3
**Proper storage of paints, lacquers and thinners in workshop**		
Yes	5	13.5
No	32	86.5
**Method of refuse disposal**		
Open dumping and burning	33	89.2
Burning in a pit	4	10.8
**Type of toilet available**		
Pit latrine	4	10.8
Pour flush	3	8.1
None	30	81.1
**PPEs sighted in workshop**		
Nose/dust mask	7	18.9
Safety boots	3	8.1
Respirator with filter	1	2.7
Waterproof overall	1	2.7
Head cover	1	2.7
Surgical mask	10	27.0

The 8-hour time weighted average concentration of total VOCs showed that total VOC was significantly higher in spray painting workshops (n = 37, mean = 13.4 ppm, standard deviation (SD) = 1.1 ppm) compared to the workplaces of controls (n = 31, mean = 0.06 ppm, SD = 0.3 ppm) (t=63.9, degrees of freedom (df)= 36, p<0.001).

The distribution of lung function test results by occupation and smoking status is shown in Table 4. The mean FVC, FVC% of predicted values, FEV1, FEV1% of predicted and FEV1/FVC were lower among spray painters, and this was statistically significant at p<0.05. The mean total serum bile acids was significantly higher among the spray painters compared with controls (p<0.001). Among painters, there was a negative correlation between years of experience and FEV1 (r=−0.22 p=0.015), as well as FEV1/FVC (r=−0.19, p=0.035).

**Table 4:** Distribution of Lung Function Test Results by Occupation and Smoking Status

Study factor	Spray painters Mean ±SD	Controls Mean ±SD	p-value
**All participants**			
Total number of subjects (n)	120	120	
FVC (liters)	3.6±0.6	3.7±0.5	**0.046**
FVC % of predicted values	93.9±10.8	96.7±8.2	**<0.001**
FEV1 (liters)	3.1±0.6	3.3±0.5	**0.002**
FEV1% of predicted values	94.6±12.2	100.3±9.1	**<0.001**
FEV1/FVC	85.5±8.7	87.9±6.2	**0.005**
**Non-smokers**			
Total number of subjects (n)	106	94	
FVC (liters)	3.7±0.6	3.8±0.44	0.087
FVC % of predicted values	94.40±10.9	96.79±8.5	0.083
FEV1 (liters)	3.1±0.6	3.3±0.5	**0.002**
FEV1% of predicted values	94.8±12.2	100.6±9.6	**<0.001**
FEV1/FVC	85.4±8.5	88.4±6.2	**0.006**
**Ever-smokers**			
Total number of subjects (n)	14	26	
FVC	3.2±0.4	3.6±0.6	**0.030**
FVC % of predicted values	89.9±9.4	96.6±7.2	**0.016**
FEV1	2.8±0.6	3.1±0.4	0.071
FEV1% of predicted values	93.4±12.3	99.0±6.7	0.069
FEV1/FVC	85.9±10.6	86.0±6.0.	0.961

Abbreviations: SD, standard deviation

The spirometry results were further analyzed based on smoking status. For non-smokers, mean FEV1 and FEV% of predicted and FEV1/FVC were lower among spray painters, and this was statistically significant. However, the mean FVC and FVC% of predicted were not significantly different across the two groups.

The comparison of spirometry readings among former and current smokers revealed that the mean FVC and FEV were lower among spray painters with p-values <0.05, while the mean value of the other lung function tests were not significantly different between spray painters and controls.

In all, 215 participants (111 spray painters and 104 controls) volunteered for TSBA assessment. The TSBA value for spray painters was 8.7±3.4 μmol/l and 4.7±2.12 μmol/l for controls. The difference in mean TSBA values was statistically significant (p<0.001).

The prevalence of lung function abnormalities among spray painters and controls was also compared. Six (5%) spray painters had restrictive lung impairment, while no electronic technician had restrictive impairment (likelihood ratio= 8.472, df = 1, p = 0.004). In addition, 10 (8.3%) spray painters had obstructive impairment, while only one (0.8%) electronic technician had obstructive impairment (χ^2^ = 7.717, df = 1, p = 0.005).

## Discussion

This study assessed spray painting workshop conditions, lung function and total bile acid levels of spray painters and controls. Nigeria has no national recommendations for spray painting workshop size. However, the Occupational Safety and Health Administration recommends a minimum space of 0.91 m around vehicles being sprayed.^30^ Therefore, a minimum booth size of 22.3 m^2^ was derived, since an average car has an approximate length of 4.2 m, breadth of 1.8 m and height of 1.7 m. Using this standard, 7 out of 10 booths were considered adequate in terms of space. However, almost all workshops had bare earth as the floor material. This is below the standard for designing and constructing spraying booths which stipulates that flooring material be made of a non-permeable, easy to clean surface.^30^ Having bare earth as the floor allows for unmitigated soil contamination by chemical agents used in spray painting. Most workshops also had walls made of materials that are not easy to clean, such as wood and tarpaulin.

None of the workshops had mechanical ventilation or independent exhaust as recommended in spray painting workshops. Similarly, most of the workshops had no windows to allow for optimal natural ventilation. Thirty-six workshops (97.3%) had no ceiling. In consideration of economic costs and a desire to aid rapid drying of painted vehicles, many small/medium scale painters prefer not to install ceilings. However, absence of a ceiling increases temperature in the workshop with an attendant increase in volatility and thus exposure to volatile organic compounds. Another practice that was frequently observed in the surveyed workshops was the use of retractable tarpaulins to prevent dust from settling on the freshly painted surfaces. A consequence of the practice of using retractable tarpaulin at workshop entrances is that there is reduced ventilation and a further increase in ambient temperature due to lack of a ceiling, likely worsening organic solvent exposures for spray painters.

Several other unsafe practices and conditions were also observed. For example, only one in eleven workshops had changing rooms, and only one had an eating area. These conditions have health implications, as contamination of food and clothing may easily occur in the workplace. Furthermore, improper storage of paints and other flammable solvents coupled with the absence of fire extinguishers make the workshops vulnerable to fire hazard. The standard safety procedure is that all spray paint workshops should have fire extinguishers due to the fire risks associated with the use of organic solvents.^30^ In addition, open dumping and burning of refuse was the most common method of waste disposal across the workshops. These dump sites are close to the workshops and highly inflammable wastes such as containers with dried paints and lacquer are exposed to naked flame. Neither hand washing facilities nor first aid kits were available in the workshops and this also poses significant sanitation and safety risks.

Total volatile organic compounds was significantly higher in spray painting workshops compared to electronic technicians' workshops. This was clearly in excess of the national standards that stipulate 1.9 ppm as the permissible limit for total VOCs in a workplace environment.^29^ It should be noted that electronic technicians do not usually use organic solvents in the course of their daily jobs in contrast to spray painters. The 8-hour time weighted average of 13.4 ppm is lower than the 41 ppm level reported by Molgaard et al. in a survey conducted in Finland to assess VOC concentrations in small-scale workshops.^31^ This may be due to the fact that continuous sampling was performed in Finnish workshops throughout working hours, whereas workshops in the present study were assessed for VOCs for a 30 minute period while painting was performed and then a time weighted average was derived. In addition, painting occurred throughout the work period in the study by Molgaard, while painting occurred less frequently in the workshops that were assessed in the present study. Nonetheless, it is noteworthy that our study is the first to document ambient concentration of total VOCs across spray painting workshops in Nigeria.

Spray painters performed significantly lower on lung function tests compared with controls. In addition, FVC and FEV1 were markedly different between the two groups and this is similar to the findings of Numan in a study conducted among 33 painters in Baghdad, Iraq which reported significant differences in FEV1 and FVC between exposed and non-exposed groups.^32^ Likewise, Metwally et al. reported lower FVC and FEV1 among a group of painters in Cairo, Egypt compared to controls.^33^ In addition, Ould-Kadi et al. reported significantly lower FEV1 spirometry readings among 106 painters in Algeria who were exposed to organic solvents compared with other workers who were exposed to welding fumes, organic dust and wood dust, as well as non-exposed individuals.^34^ The negative correlation between years of experience and FEV1 and FEV1/FVC readings may suggest an inverse relationship between spirometry and time exposure, which is similar to the findings of Metawally and colleagues in Cairo.^33^ However, some studies have demonstrated no relationship between lung function status and solvents exposure.^35^,^36^ For instance, Ernstgard et al. found no effect of exposure on the pulmonary functions among a group of 10 painters and controls, but this may be due to the use of water-based solvents among the surveyed painters and the small sample size.^36^ In addition, in a study of car painters in Tamilnadu, India, Revathi and Chandrasekhar found no significant difference in FEV1 and FVC readings between exposed and control groups.^37^ A major reason for this result was that the study was conducted among young men who had worked between 1–5 years as painters with presumably low solvents exposure. However, spray painters had significantly more obstructive and restrictive ventilatory impairments than controls.

In view of the possibility that smoking may affect lung function status, data were further analyzed based on smoking status. For non-smokers, FEV and FEV1/FVC were significantly different between spray painters and controls, whereas only FVC and %FVC predicted were significantly different among painters and controls who were ever-smokers.

In the present study, total serum bile acids was used as a proxy for organic solvents exposure. The almost two-fold higher average value of TSBA among painters compared to controls indicates that TSBA is an effective biomarker for assessing groups of solvent-exposed workers, as suggested in the literature.^16^,^38^ This was similar to findings from a study of 57 Egyptian workers exposed to organic paints, where assessment of serum total bile acids was the only liver function test that was affected in the exposed group without an increase in the frequency of impaired liver-related symptoms, even at short duration of exposure or with low exposure levels.^15^ A limitation of the present study was that personal exposure to VOCs could not be quantified, as personal gas samplers were not available. However, the use of a sensitive VOC gas monitor and air sampler in the breathing zone of workers provided a useful estimate of total VOCs in the workshops. Serious respiratory effects (mainly occupational asthma) that may occur among spray painters working in car body repair workshops are generally caused by the diisocyanate hardener (mainly hexamethylene diisocyanate) used in two-component polyurethane paint systems. The present study did not assess the presence of isocyanates in workshops.

## Conclusions

Spray painters in Nigeria work in hazardous settings due to the chemicals they work with, the particular environmental conditions in their workshops and poor use of PPEs. Spray painters had significantly lower spirometry test results and elevated TSBA compared to controls. Concentrations of total VOCs across the workshops were clearly in excess of national standards. Future studies to assess individual exposure levels to organic solvents using personal air samplers or bio-monitoring is recommended. More needs to be done in terms of workplace regulation and enforcements to ensure that spray painters comply with minimum standards of workplace hygiene and sanitation. Although relatively expensive, the introduction of water-based solvents into the Nigerian automobile spraying industry should be encouraged as they are associated with fewer chemical hazards and harmful effects. The provision of standard spraying booths with mechanical ventilation and exhaust systems to minimize organic solvent exposure is an appropriate intervention to safeguard the health of these workers.
